# Alternative Bio-Based Solvents for Extraction of Fat and Oils: Solubility Prediction, Global Yield, Extraction Kinetics, Chemical Composition and Cost of Manufacturing

**DOI:** 10.3390/ijms16048430

**Published:** 2015-04-15

**Authors:** Anne-Gaëlle Sicaire, Maryline Vian, Frédéric Fine, Florent Joffre, Patrick Carré, Sylvain Tostain, Farid Chemat

**Affiliations:** 1 GREEN (Groupe de Recherche en Eco-Extraction de Produits Naturels), Université d’Avignon et des Pays de Vaucluse, INRA, UMR 408, 84000 Avignon, France; E-Mails: anne-gaelle.sicaire@univ-avignon.fr (A.-G.S.); farid.chemat@univ-avignon.fr (F.C.); 2 CETIOM (Centre Technique Interprofessionnel des Oléagineux et du Chanvre industriel), 11 rue Monge, 33600 Pessac, France; E-Mail: fine@cetiom.fr; 3 ITERG (Institut des Corps Gras), 11 rue Monge, 33600 Pessac, France; E-Mail: f.joffre@iterg.com; 4 CREOL (Centre de Recherche et d’Expérimentation sur les oléagineux), 11 rue Monge, 33600 Pessac, France; E-Mail: carre@cetiom.fr; 5 SAIPOL (Société Agro Industrielle de Patrimoine Oléagineux), Boulevard Maritime, 76530 Grand-Couronne, France; E-Mail: s.tostain@saipol.fr

**Keywords:** alternative solvent, solvent selection, bio-based solvent, MeTHF, extraction, kinetic parameters, economic evaluation

## Abstract

The present study was designed to evaluate the performance of alternative bio-based solvents, more especially 2-methyltetrahydrofuran, obtained from crop’s byproducts for the substitution of petroleum solvents such as hexane in the extraction of fat and oils for food (edible oil) and non-food (bio fuel) applications. First a solvent selection as well as an evaluation of the performance was made with Hansen Solubility Parameters and the COnductor-like Screening MOdel for Realistic Solvation (COSMO-RS) simulations. Experiments were performed on rapeseed oil extraction at laboratory and pilot plant scale for the determination of lipid yields, extraction kinetics, diffusion modeling, and complete lipid composition in term of fatty acids and micronutrients (sterols, tocopherols and tocotrienols). Finally, economic and energetic evaluations of the process were conducted to estimate the cost of manufacturing using 2-methyltetrahydrofuran (MeTHF) as alternative solvent compared to hexane as petroleum solvent.

## 1. Introduction

Solvents are usually volatile organic compounds (VOCs) sourced from non-renewable resources. They are also usually harmful to health and the environment. Currently, hexane is the most used solvent for extraction of vegetable oils for its various qualities such as ease of removal by evaporation from the products, convenient boiling point (high enough to limit losses during extraction but sufficiently low to limit heat consumption during its recovery), stability and ideal functionalities in terms of lipid selectivity [1]. Research of greener, biodegradable and non-dangerous solvents has become a major concern for academic and industrial research considering the fact that *n-*hexane, one of the main constituents of extraction grade hexane, is sourced from fossil resources and registered under the REACH Regulation as a category 2 reprotoxic and as a category 2 aquatic chronic toxic. Green solvents have to satisfy the principles of Green Chemistry and in this context, bio-based derived chemicals show a great potential.

**Figure 1:**
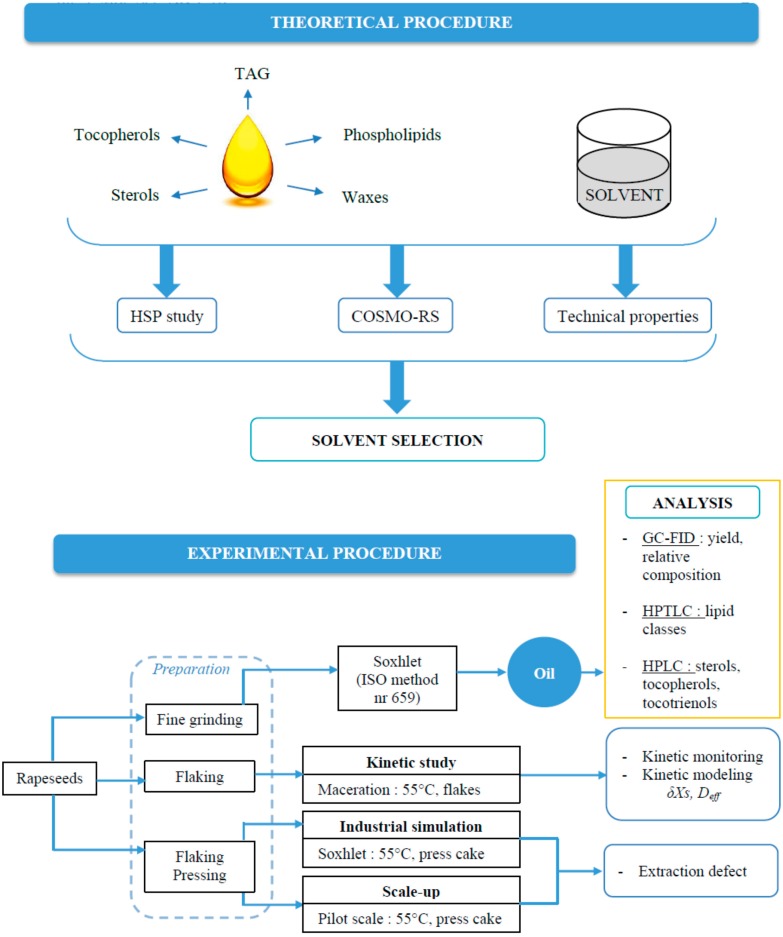
Scientific approach (TAG: triglycerides; COSMO-RS: COnductor-like Screening MOdel for Realistic Solvation.).

We begin our study with the Hansen Solubility Parameters study [2] to evaluate and understand the interactions between alternative solvents and major constituents of vegetable oils (triglycerides (TAGs), sterols, tocopherols, phospholipids, waxes) as can be seen in Figure 1. We tried another solubility study using the COnductor-like Screening MOdel for Realistic Solvation (COSMO-RS) simulation [3,4] with some of the major TAGs, sterols and tocopherols found in rapeseed oil in order to assess their relative solubility within solvents. Both simulations show that 2-methyltetrahydrofuran (MeTHF) [5,6], appears to be a promising alternative to *n-*hexane for the extraction of vegetable oils. MeTHF, produced from biomass like corncobs, sugar cane bagasse or oat hulls, is one of these “green” and bio-based solvents. It can be synthesized from levulinic acid or furfural produced from C5 and C6 sugars of cellulose and can be degraded by solar light and air [7]. The relevant properties of MeTHF [8,9] compared to *n-*hexane are listed in Table 1. The most important properties are the apolar aprotic chemical characteristic of this solvent but also the boiling point and evaporation heat of MeTHF, which are similar to hexane. Surprisingly, up to now, only two applications of MeTHF as extraction solvent were found in the literature. Vom Stein *et al.* [10] described the fractionation of lignocellulose using MeTHF as solvent with high yield and selectivity. Saunois *et al.* [11], from Laboratoires Expanscience, patented extraction of oil with high unsaponifiable content from biomass such as dehydrated avocado and lyophilized avocado powder using MeTHF as bio-based solvent.

Laboratory experimental study has been conducted with MeTHF and *n-*hexane for the extraction of rapeseed oil to assess the performances of the solvent in terms of global yields, kinetic and diffusion studies, and detailed composition of vegetable oil. The study has been completed by transposition to pilot plant experimentation not only to have the expected solvent performance, yield and composition but also to see the real technical problems that generally occur at industrial scale. The experimental procedure has been conducted as shown in Figure 1. The objectives of this work were also to obtain the technical data in order to perform an economic and energetic feasibility analysis for the recovery of vegetable oil from crops using MeTHF as a bio-based solvent and to compare it with hexane as a non-renewable solvent.

**Table 1:** Properties of *n*-hexane and MeTHF.

Solvent	*n*-Hexane	MeTHF
Chemical structure	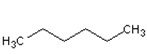	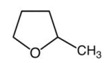
Molecular formula	C_6_H_14_	C_5_H_10_O
Molecular weight (g·mol^−1^)	86.18	86.13
Density (25 °C, g·cm^−3^)	0.675	0.855
Flash point (°C)	−23	−11.1
Boiling point (°C)	69	80
Viscosity (25 °C, Cp)	0.31	0.60
Enthalpy of vaporization (kj·kg^−1^)	328	375
Miscibility in water	0.001 g/100 g	4.1 g/100 g
Dielectric constant (20 °C)	1.87	6.97
Hansen Solubility Parameters (cal^1/2^·cm^−3/2^)		
δ^a^	7.3	8.9
δ_d_	7.3	8.3
δ_p_	0	1.9
δ_h_	0	3.0

## 2. Results and Discussion

### 2.1. COSMO-RS and Hansen Assisted Solvent Selection

Hansen prediction gives an evaluation of the ability of a solvent to dissolve major components of rapeseed oil [12] (TAGs, phospolipids, sterols, tocopherols, wax). Several solvents (*n-*hexane, MeTHF, *d-*limonene, *p-*cymene, methylacetate, ethylacetate, butanol, isopropanol (IPA) and ethanol) were selected for the simulation. The ability of a solvent to dissolve solutes is evaluated by the relative energy difference (RED) calculated by the software. Table 2 shows the RED calculated for the selected solvents with TAGs, phospholipids, tocopherols, sterols and wax. As can be seen, for *n-*hexane RED > 1 which means that in fact *n-*hexane is not the best solvent from a theoretical perspective for the extraction of major compounds except W (wax C48) but waxes are not desirable compounds in oils. Regarding these results, terpenes (*p-*cymene and *d-*limonene) and MeTHF seem to be good solvents for the extraction of TAGs as RED < 1; they are better than *n-*hexane for the solvation of phospholipids, tocopherols and sterols. Considering wax, among these three solvents, only MeTHF theoretically avoids the extraction as RED > 1. Other solvents (methylacetate, ethylacetate, butanol, IPA and ethanol) are theoretically good solvents only or solvation of phospholipids.

A COSMO-RS simulation was also conducted in order to determine the relative solubility of four major TAGs, two tocopherols and three sterols of rapeseed oil in the various studied solvents. The software integrates a quantic chemistry approach; it permits the calculation of various properties such as the relative solubility of a compound in several solvents as presented in Table 3. As the logarithm of the best solubility is set to 0 and all other solvents are given relatively to the best solvent, it can be noticed that at 55 °C, which is the temperature of extraction under industrial conditions, log (x-solub) for three TAGs with *n-*hexane (taken as the reference) is equal to 0. This means that it is the best solvent compared to other tested solvents. However, log(x-solub) for *d-*limonene, *p-*cymene, MeTHF and ethylacetate are also set to 0; in terms of relative solubility these four solvents are equivalent to *n-*hexane for the solubilization of major TAGs of rapeseed oil. Concerning the tocopherols, the 3 alcohols (butanol, IPA and ethanol) can be discarded as their relative solubilities are worse than in *n-*hexane. The three best solvents for tocopherols extraction are MeTHF, methylacetate and ethylacetate. Regarding the sterols only *p-*cymene and ethanol are not as effective as *n-*hexane for the extraction of S1 and S2 (campesterol and β-sitosterol).

**Table 2:** The relative energy difference (RED) values for HSP assisted selection of alternative solvent to *n*-hexane for the extraction of rapeseed oil.

Items	δD	δP	δH	TAG1	TAG2	TAG3	TAG4	PE	PC	LPC	T1	T2	S1	S2	S3	W
***n-*hexane**	14	0	0	1.12	1.10	1.06	1.09	2.96	2.74	3.67	1.09	1.40	1.43	1.36	1.56	0.81
**MeTHF**	16.4	4.7	4.6	0.83	0.92	0.97	0.89	1.32	1.23	2.09	1.06	0.82	0.90	0.92	0.99	1.15
***d-*limonene**	16.7	1.8	3.1	0.15	0.16	0.21	0.14	2.00	1.80	2.71	0.24	0.23	0.25	0.20	0.40	0.44
***p-*cymene**	17.3	2.3	2.4	0.49	0.38	0.39	0.43	2.13	1.97	2.83	0.50	0.18	0.24	0.23	0.29	0.65
**Methylacetate**	15.5	7.2	7.6	1.83	1.95	2.00	1.91	0.67	0.82	1.26	2.06	1.88	1.94	1.96	2.01	2.13
**Ethylacetate**	15.8	5.3	7.2	1.40	1.53	1.59	1.48	0.71	0.62	1.42	1.61	1.46	1.50	1.53	1.58	1.72
**Butanol**	16	5.7	15.8	3.35	3.49	3.54	3.43	1.57	1.59	1.00	3.48	3.36	3.35	3.40	3.37	3.67
**IPA**	15.8	6.1	16.4	3.53	3.67	3.72	3.61	1.72	1.76	1.12	3.66	3.55	3.54	3.59	3.56	3.85
**ethanol**	15.8	8.8	19.4	4.47	4.66	4.60	4.55	2.53	2.67	1.83	4.61	4.47	4.47	4.51	4.48	4.79

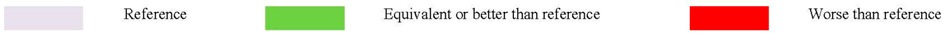

(TAG 1 (R1: C18:3*n* − 3, R2: C18:2*n* − 6, R3: C18:2*n* − 6), TAG 2 (R1: C18:2*n* − 6, R2: C18:1*n* − 9, R3: C18:1*n* − 9), TAG 3 (R1: C18:1*n* − 9, R2: C18:1*n* − 9, R3: C18:1*n* − 9), TAG 4 (R1: C18:1*n* − 9, R2: C18:2*n* − 6, R3: C18:2*n* − 6), PE (phosphatidylethanolamine R1: C18:1*n* − 9, R2: C18:2*n* − 6), PC phosphatidylcholine R1: C18:1*n* − 9, R2: C18:2*n* − 6), LPC (lysophosphatidylcholine R: C18:2*n* − 6), T1 (α-tocopherol), T2 (γ-tocopherol), S1 (campesterol), S2 (β-sitosterol), S3 (brassicasterol), W (wax C48)).

**Table 3:** COSMO-RS assisted solvent selection: relative solubility (log (x-solub)) of major compounds of rapeseed oil in several solvents at 55 °C.

	TAG1	TAG2	TAG3	TAG4	T1	T2	S1	S2	S3
***n-*hexane**	−0.3797	0	0	0	−0159	−0.1363	−0.4468	−0.4773	−0.4940
**MeTHF**	0	0	0	0	0	0	0	0	0
***d-*limonene**	0	0	0	0	0	−0.0229	−0.4030	−0.4162	−0.4023
***p-*cymene**	0	0	0	0	−0.0040	−0.0787	−0.4805	−0.4842	−0.4556
**Methylacetate**	0	−0.0463	−0.5269	−0.2164	0	0	−0.2423	−0.2101	−0.1229
**Ethylacetate**	0	0	0	0	0	0	0	0	0
**Butanol**	−0.7385	−1.0072	−0.9738	−1.1775	−0.4340	−0.3684	−0.1987	−0.1645	−0.1540
**IPA**	−0.7811	−1.1702	−1.1342	−1.3628	−0.4607	−0.7938	−0.2375	−0.1996	−0.1789
**ethanol**	−1.4208	−2.0056	−1.9840	−2.2436	−0.8844	−0.1363	−0.5411	−0.4857	−0.4508

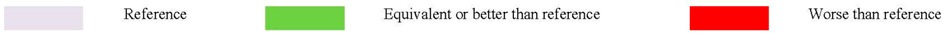

(TAG 1 (R1: C18:3*n* − 3, R2: C18:2*n* − 6, R3: C18:2*n* − 6), TAG 2 (R1: C18:2*n* − 6, R2: C18:1*n* − 9, R3: C18:1*n* − 9), TAG 3 (R1: C18:1*n* − 9, R2: C18:1*n* − 9, R3: C18:1*n* − 9), TAG 4 (R1: C18:1*n* − 9, R2: C18:2*n* − 6, R3: C18:2*n* − 6), T1 (α-tocopherol), T2 (γ-tocopherol), S1 (campesterol), S2 (β-sitosterol), S3 (brassicasterol)).

However, various parameters other than solubility have to be considered; in fact data such as melting point, viscosity, boiling point and energy required for solvent evaporation as well as log P or toxicity category, are technical properties of the solvent that are important for the solvation of specific components but also for the implementation of the process at different scales.

**Figure 2:**
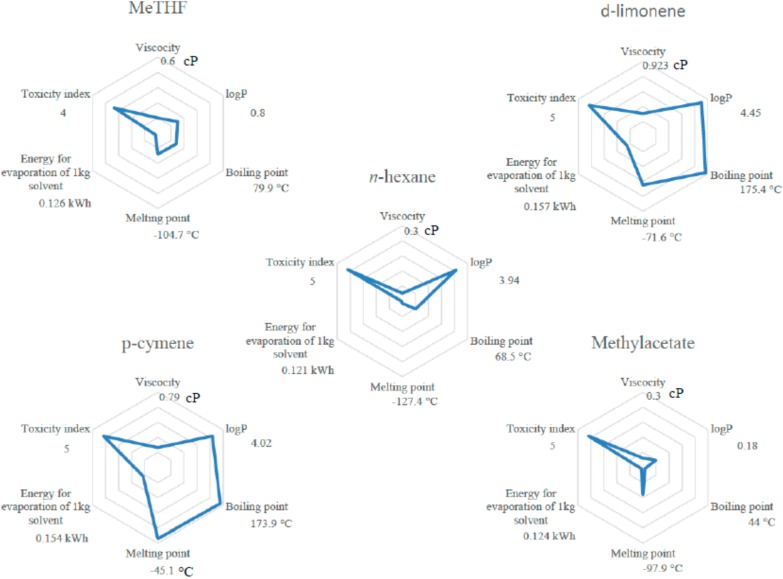

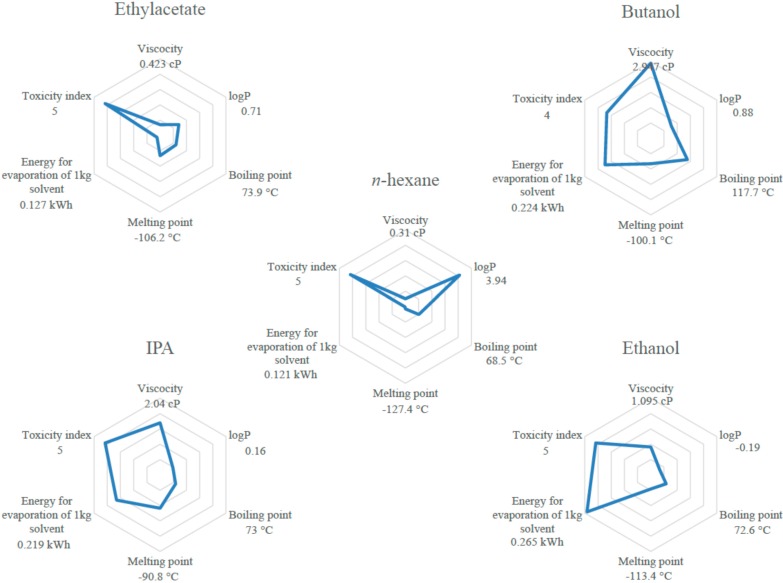
Properties of solvents *vs.* *n*-hexane.

Melting point has to be higher than 25 °C in order to be liquid at ambient temperature. Energy for solvent evaporation (dependent on boiling point) has to be as low as possible. Those properties are represented in Figure 2 and give a global view of the potential of the tested solvents for the substitution of *n-*hexane. Among all these properties, energy for solvent evaporation for *d-*limonene, *p-*cymene, butanol, IPA and ethanol is much higher than for *n-*hexane. Regarding the technical properties, tested alcohols (butanol, IPA and ethanol) and terpenes (*p-*cymene and *d-*limonene) are discarded for the substitution.

Considering these theoretical and technical approaches, MeTHF can be considered as the best alternative to *n-*hexane among all other tested solvents as it has good solubilization abilities regarding desirable compounds in oil. Moreover most of its technical properties are not significantly different from *n-*hexane especially the energy required for solvent evaporation. MeTHF was then tested experimentally to confirm these theoretical predictions.

### 2.2. Solvent Comparison: Total Lipid Yield and Composition

**Figure 3:**
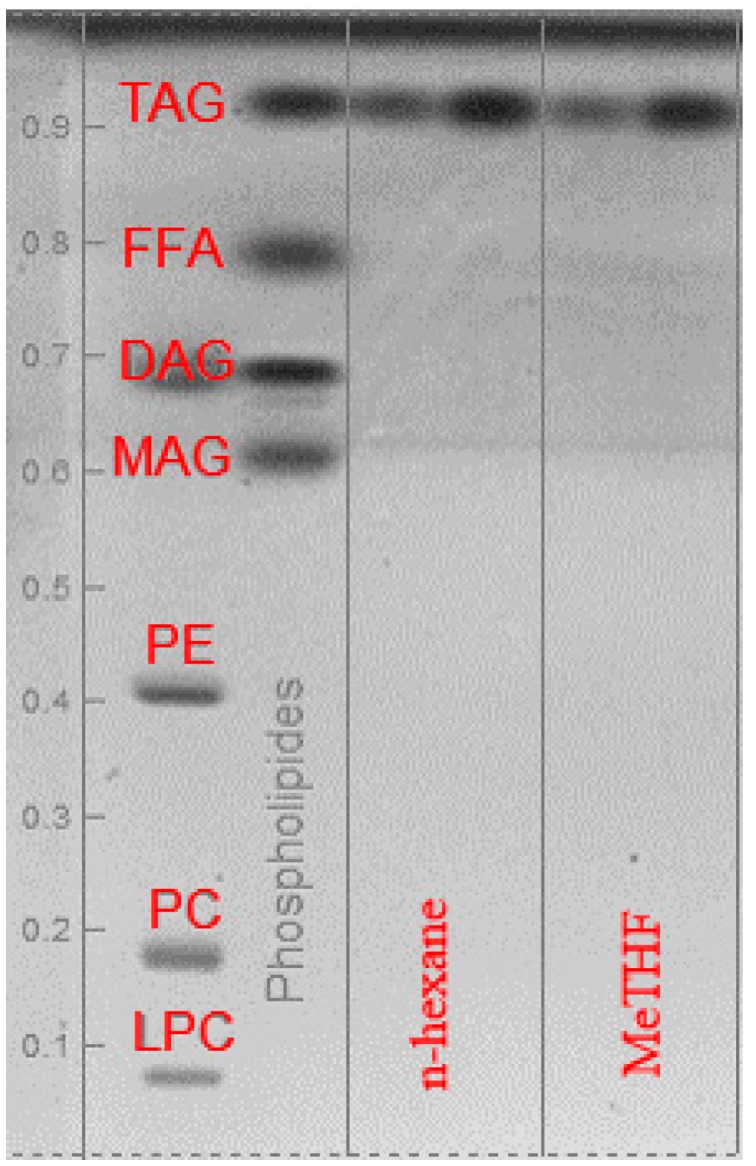
HP-TLC plate: lipid classes in rapeseed oil extracted with *n-*hexane and MeTHF (TAG: triglyceride; FFA: free fatty acids; DAG: diglyceride; MAG: monoglyceride; PE: phosphatidylethanolamine; PC: phosphatidylcholine; LPC: lysophosphatidylcholine).

After 8 h Soxhlet extraction (standard ISO 659), relative composition and total lipid yield of the extracts were determined by GC-FID after transmethylation of fatty acids. As shown in Table 4, the lipid yield and lipid profile of oils obtained from MeTHF extraction is comparable to the one extracted with hexane extraction. No significant selectivity between *n-*hexane and MeTHF was noted as the composition in fatty acids remains the same. The main fatty acids in extracted oils are oleic (C18:1), linoleic (C18:2), linolenic (C18:3) and palmitic (C16:0) which represent more than 90% of the total fatty acids in extracted oil. Moreover, a HPTLC analysis, see Figure 3, provides confirmation that more than 99% of the constituents extracted with both solvents are triglycerides (TAG).

**Table 4:** Extraction yield and fatty acid composition of rapeseed oil extracted with *n-*hexane and MeTHF.

Fatty Acids	Extracted Amounts of Fatty Acids (%)	
	*n*-Hexane	MeTHF
C16	4.44 ± 0.23	4.45 ± 0.18
C16:1 *n* − 7	0.24 ± 0.01	0.27 ± 0.02
C18	1.37 ± 0.07	1.34 ± 0.08
C18:1 *n* − 9	58.26 ± 0.88	58.28 ± 0.83
C18:2 *n* − 6	22.59 ± 0.32	22.81 ± 0.27
C18:3 *n* − 3	9.43 ± 0.13	9.33 ± 0.17
C20	0.45 ± 0.01	0.46 ± 0.04
C20:1 *n* − 9	1.64 ± 0.09	1.60 ± 0.05
C20:5 *n* − 3	0.22 ± 0.01	0.28 ± 0.01
C22:1 *n* − 9	0.22 ± 0.01	1.68 ± 0.06
C22:2 *n* − 6	0.22 ± 0.01	0.58 ± 0.03
∑SFAs	6.26	6.25
∑MUFAs	61.80	61.83
∑PUFAs	32.75	33.00
Extraction yield (g/100 g DM)	46.34 ± 0.48	45.96 ± 0.80

SFA: Saturated Fatty Acid; MUFA: Monounsaturated Fatty Acid; PUFA: Polyunsaturated Fatty Acid.

Other constituents such as diglyceride (DAG), monoglyceride (MAG), free fatty acids (FFA) or phospholipids were present as traces and were not quantifiable by HP-TLC. Lipid profile of the oil as well as the compositions in terms of lipid classes are the same using *n-*hexane or MeTHF.

The micronutrient content of the oil was determined by HPLC analysis as shown in Table 5 and Table 6. The main constituents of unsaponifiables are tocopherols, which are known as natural antioxidants, sterols and tocotrienols. No significant difference in tocopherol content was found between oils extracted with *n-*hexane or MeTHF. Tocotrienols are below the quantification limit for both types of samples. β-Sitosterol, campesterol and brassicasterol are identified as the three major sterols in extracted oil. They occur in extracted oils in the same proportions as shown in Table 5. 

**Table 5:** Content of sterols in extracted rapeseed oils with *n*-hexane and MeTHF.

Sterols	*n*-Hexane (%)	MeTHF (%)	
Cholesterol	0.33 ± 0.06	0.40 ± 0.00	
Brassicasterol	8.03 ± 0.06	8.20 ± 0.10	
24 methyl-cholesterol	1.23 ± 0.12	1.20 ± 0.00	
Campesterol	37.47 ± 0.06	37.23 ± 0.31	
Campestanol	0.10 ± 0.00	0.13 ± 0.12	
Stigmasterol	0.20 ± 0.00	0.27 ± 0.06	
δ7-Campesterol	0.27 ± 0.06	0.27 ± 0.06	
δ5.23 Stigmastadienol	0.23 ± 0.06	0.23 ± 0.06	
Clerosterol	0.60 ± 0.00	0.57 ± 0.06	
β-sitosterol	48.30 ± 0.30	48.30 ± 0.26	
Sitostanol	0.20 ± 0.00	0.20 ± 0.00	
δ5-Avenasterol	2.20 ± 0.10	2.37 ± 0.06	
δ5.24 Stigmastadienol	0.50 ± 0.00	0.53 ± 0.06	
δ7-Stigmasterol	0.10 ± 0.00	<0.1	
δ7-Avenasterol	0.25 ± 0.21	<0.1	
Unidentified	<0.1	<0.1	
Sterols in extracted oils (mg/100 g)	881 ± 14	810 ± 22	

For the standard parameters studied, oil extracted with MeTHF is equivalent to oil extracted with *n-*hexane in both qualitative and quantitative compositions.

**Table 6:** Content of tocopherols and tocotrienols in extracted rapeseed oils with *n*-hexane and MeTHF.

Items	*n*-Hexane (mg/kg Fat)	MeTHF (mg/kg Fat)
Tocopherol		
Acetate	<5	<5
α	292 ± 10	277 ± 28
β	3.3 ± 0.6	3.5 ± 0.7
γ	488 ± 10	443 ± 13
δ	14.3 ± 0.58	13.3 ± 0.6
Term vitamin E (TE/100 mg)	34.2 ± 1.0	32.3 ± 2.7
Tocotrienols		
α	<2	<2
β	<2	<2
γ	<2	<2
δ	<2	<2
Total (tocopherol + tocotrienol) (mg/kg fat)	797 ± 119	735 ± 110

### 2.3. Kinetic Study: Good Diffusion of MeTHF

Solvent extraction occurs in two stages; a first solvent-exchange surface interaction takes place for a short time-frame. Thus, starting accessibility δXs (in g of extract/g of dry material) reveals the amount of extract obtained in a very short time-frame (*t* near 0) through the convection of solvent interacting with the exchange surface. Afterward, the main part of the operation is controlled through various penetration processes of the solvent within the material (capillarity, molecular diffusivity, *etc.*). The driving force of the global operation is the gradient of concentration and the model can be similar to Fick’s Law with an effective diffusivity (*D*_eff_) (m^2^·s^−1^) as the process coefficient [13,14]. The solutions required for this diffusion equation are dependent on initial conditions. Crank’s solution [15] is described for a sphere, the hypothesis for the particles, as it is a function of the geometry of the product. Experimental data close to initial time have to be excluded to identify *D*_eff_ and should only concern data for *t* > *t*_0_ [16].

1st Fick’s Law [14]:

(1) $$\frac{{\text{ρ}}_{\text{s}}}{{\text{ρ}}_{\text{d}}}\left({\vec{v}}_{\text{s}}-{\vec{v}}_{\text{d}}\right)=-D_{\text{eff}}\vec{\nabla }\left(\frac{{\text{ρ}}_{\text{s}}}{{\text{ρ}}_{\text{d}}}\right)$$

One can assume the absence of expansion or shrinkage of the solid particles which are not moving, *i.e.*, ${\vec{v}}_{\text{d}}$ = 0 and ${\text{ρ}}_{\text{d}}$ = constant.

(2) $${\text{ρ}}_{\text{s}}{\vec{v}}_{\text{s}}=-D_{\text{eff}}\vec{\nabla }\;{\text{ρ}\;}_{\text{s}}$$

Crank’s solution for a sphere:

(3) $$\frac{X_{\infty }-X}{X_{\infty }-X_{0}}=\overset{\infty }{\underset{i=1}{\sum }}\frac{6}{i^{2}{\text{π}}^{2}}exp\left(-\frac{i^{2}{\text{π}}^{2}D_{\text{eff}}}{r_{\text{d}}^{2}}(t-t_{0})\right)$$

(4) $$\frac{X_{\infty }-X}{X_{\infty }-X_{0}}=Aexp\left(-k\left(t-t_{0}\right)\right)$$

(5) $$Ln\left(\frac{X_{\infty }-X}{X_{\infty }-X_{0}}\right)=-k\left(t-t_{0}\right)$$

(6) $$D_{\text{eff}}=k\frac{r_{\text{d}}^{2}}{{\text{π}}^{2}}$$

Starting accessibility corresponds to the value obtained by extrapolating diffusion model to *t* = 0: *X*_0_ ≠ (*X_i_* = 0):

(7) $$X_{0}-X_{i}=X_{0}=\;\text{δ}X_{\text{s}}$$

with δ*X*_s_ or *X*_0_: Starting accessibility (g of extract/g of dry material), amount of solute available at the surface of the matrix;

*t*_0_: Extraction time corresponding to *X*_0_ (min);
*D*_eff_: Effective diffusivity (m^2^·s^−1^);
ρ_s_: Apparent density of the solute within the solid matrix (kg·m^−3^);
ρ_d_: Apparent density of the solid dry material (kg·m^−3^);
*v*_s_: Velocity of the solute (m·s^−1^);
*v*_d_: Velocity of the solid dry material (m·s^−1^);
$X_{\infty }$: Amount of solute within the matrix (mg·g^−1^ dry material);
*r*_d_: Radius (m);
*X*: Amount of solute extracted at time (*t*) (mg·g^−1^ dry material);
*k*: Transfer coefficient (m·s^−1^).

**Figure 4:**
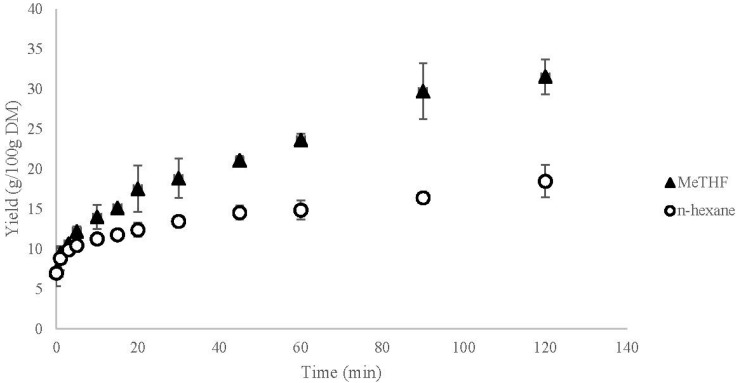
Extraction yield of rapeseed oil at 55 °C with *n*-hexane and MeTHF.

Kinetics of the extractions of coarsely ground rapeseeds with *n-*hexane and MeTHF are represented in Figure 4.

Starting accessibility and diffusivity were calculated using Equations (5) and (6). Starting accessibility is determined by extrapolating the value for *t* = 5 min at *t* = 0 which means *t*_0_ = 5 min. Starting accessibility is 0.100 g/g DM for the extractions with both *n-*hexane and MeTHF which represents 21% of total oil available in the matrix. This value, *X*_0_, is equivalent for both solvents which is in accordance with the theory of solvation of the oil directly available at the surface of the matrix.

The *D*_eff_, effective diffusivity coefficient is calculated thanks to Equation (6) with *X*_0_ = 0.100 g/g DM and *r*_d_ = 1 mm. *X*_0_ is experimentally obtained by the kinetic monitoring presented Figure 4 and *r*_d_ is obtained by flake sieving. The value of *D*_eff_ with *n-*hexane as solvent is 0.034 × 10^−10^ and 0.122 × 10^−10^ m^2^·s^−1^ with MeTHF. This effective diffusivity coefficient *D*_eff_ translates the speed with which the compound is extracted from the matter. Using MeTHF allows for an improvement by a factor 3.5 of the internal diffusion of oil and most probably for a faster extraction than with *n-*hexane.

### 2.4. Industrial Simulation

On the basis of the previous results and in order to reproduce what occurs during industrial processing for a further pilot transposition, Soxhlet extractions were performed at 55 °C with a special double jacket modified Soxhlet apparatus.

Rapeseeds were initially pressed in order to obtain cake corresponding to partially defatted meal (oil content: 18.22%, water content: 7.85%). The cake was subsequently extracted by either hexane or MeTHF. The extraction was followed thanks to kinetic monitoring and was considered completed after 2 h with the appearance of a plateau. After the extractions, residual oil in the matrix was investigated. The extraction with *n-*hexane implies a residual oil content of 1.06% where MeTHF only left 0.6%. At least, in the same extraction conditions MeTHF allows for a better oil extraction than *n-*hexane. This may have a positive economic impact with a better return from the oil extracted from the matrix.

### 2.5. Pilot Scale up

Hexane and MeTHF were then tested on press cake (see Section 2.4.) using the 6 L percolation pilot extractor at Centre de Recherche et d’Expérimentation sur les Oléagineux (CREOL), Pessac. The extraction was conducted by doing five washings of 30 min with 1.5 kg of solvent per kg of cake at 55 °C. A kinetic monitoring was realized (not reported here); the same kinetic trends than the lab ones are observed. After the extractions, the residual oil percentage in the meal for hexane is 1.8% and 0.8% for MeTHF, which is consistent with the percentage determined after the table-top extractions. At this pilot scale MeTHF allows for an additional 1% of extracted oil compared to hexane, which represents around 10 kg/t seeds. CETIOM quality surveys [17] indicate that industry standards leave, on average, about 3.1% of oil in the meal [18]. On the basis of 550 kg of meal per tonne of seeds it represents 16.5 kg/t seeds. By extrapolating the improvement of the yield one could expect to only leave 7.3 kg of oil per tonne of seeds and thus gain 9.2 kg of oil, which represents a gain of about 5 EUR/t seeds. The extraction yield is improved by using MeTHF but the extraction is also faster because only three washings are needed to extract nearly 95% of total oil whereas five washings are required with hexane to extract 96% of total oil.

The results of the pilot are consistent with the results of the table-top experiments. One could also expect that less solvent might be required with MeTHF, which would have a positive impact on the energy consumption for distillation and solvent recovery.

### 2.6. Economic and Energetic Study

#### 2.6.1. Energetic Requirements

Energy consumption is a key issue from an industrial perspective especially for the substitution of hexane in oil extraction processes, as the alternative solvent should not cause significant additional energetic costs. Based on the article of Carré [18] that gives information about energy requirements for the rapeseed crushing process (that covers both preparation and extraction) using hexane, calculations were made to determine the energy required with MeTHF in order to evaluate the energy consumption of the process and to compare it to the data for the process using hexane. Table 7 shows the consumption of the operations occurring during the process using hexane and MeTHF. The assessment of the theoretical heat required at the different stages of crushing must take into account the specific heats of seeds, oilcake, meal and oil.

**Table 7:** Energetic consumption for the total crushing process with *n*-hexane and MeTHF.

Step	Material	Change	Heat Required (MJ/t)		Steam Consumption (kg/t)	
			*n*-Hexane	MeTHF	*n*-Hexane	MeTHF
Conditioning	Seeds	T: 15 ==> 60 °C	78	78	36.6	36.6
Cooking	Flakes	T: 55 ==> 105 °C	110	110	53.4	53.4
		Vaporization of 30 kg of water	66	66	32.2	32.2
	Air (dry) for drying	T: 20 ==> 100 °C	15	15	7.2	7.2
Total preparation			270.0	270.0	129.0	129.0
Desolventization	Meal	T: 55 ==> 105 °C	52	52	26.1	26.1
	Solvent	T: 55 ==> Bp	8	13	3.9	6.3
		Vaporization	75	108	37.6	54.1
	Steam	Sparged	77	112	27.5	40.4
		Condensed	38	38	13.5	13.5
Distillation	Miscella	T: 55 ==> Bp	8	22	2.7	8.1
	Oil	T: Bp ==> 110 °C	10	7	3.6	2.7
	Solvent	Vaporization	122	139	58.9	67.3
Total distillation			139.0	168.9	65.2	78.1
Heat recovered from the gas from the DT			−104.3	−126.7	−48.9	−58.6
Total extraction			284	365	125	160
Losses (5%)			28	32	12.7	14.5
Total preparation + extraction			581	666	267	304
Total			851	936	396	433

T: Temperature; Bp: Boiling point; DT: Desolventizer.

Specific heats considered in the study are 1.9 kJ·°C^−1^·kg^−1^ for seeds and meal [18] and 2 kJ·°C^−1^·kg^−1^ for oil [18]. For hexane, specific heat is 2.27 kJ·°C^−1^·kg^−1^, latent heat of vaporization is 328 kJ·kg^−1^ and boiling point is 69 °C [8]. For MeTHF, specific heat is 1.758 kJ·°C^−1^·kg^−1^, latent heat of vaporization is 375 kJ·kg^−1^ and boiling point is 80 °C [8]. Seeds were considered having a water content of 7.8% and an oil content of 45.0%, which is in accordance with average values measured over the 2012 harvest [19]. Meal was considered to have an average water content of 11.3% and an oil content of 2.7% [20] and press cake was considered to have an oil content of 21% which are values known from several surveys [18]. The flaking temperature is 60 °C, during flaking there is a loss of 5 °C and a loss of 29.5 kg of water per tonne of crushed seeds (7.9% to 5.0% water in flakes). The extraction is performed at a temperature between 45 and 60 °C and it was fixed at 55 °C for the calculations. The solvent enters the extractor at around 40 °C and we considered a concentration of oil in miscella of 25% as well as retention of hexane by the dry matter of 30%.

Desolventation is made by indirect heating and direct steam injection. The amount of steam to be used is modulated to reach a temperature of 70 °C in the case of hexane, and 80 °C in the case of MeTHF in the vapors leaving the desolventizer. According to Schumacher [21], 0.12 kg of steam per kg of hexane is required to reach this temperature in the case of hexane meals. By extrapolating it was considered that 0.14 kg of steam per kg of MeTHF is required. The enthalpy of vaporization of condensed steam is lost during the drying step; in the study it was considered that it was consumed only the amount of steam required to reach 11.5% of water in meal. For the miscella distillation, there is heat recovery from the desolventizer. The gas that comes out the desolventizer passes through an exchange column to recover the condensation heat and evaporates a portion of the hexane from the miscella. This exchange according to Anderson [22] provides 75% of the heat necessary for distillation of the miscella. Steam used for desolventization is 10 bar saturated steam and six bar saturated steam for distillation. The energy needs for the preparation step do not vary from one case to another because no solvent is used. However for the extraction step, as the boiling point is higher for MeTHF than for hexane, more energy is required to attain the boiling temperature as well as more direct steam is required for stripping the residues. The same observation is made for the distillation of the miscella with small savings on the heating of the oil with MeTHF compared to hexane because it was considered that less solvent can be used thanks to better diffusivity. In fact, extraction at higher temperature might be achieved by using the excess heat from the cake to preheat the solvent, and therefore the cake does not require a lot of cooling before extraction so a little heat will be saved for distillation. More interestingly, maintaining higher temperature between pre-press and extractor could avoid a possible resumption of the phospholipase activity and, in consequence, result in lower non-hydratable phosphorus concentration in extraction. The total amount of energy assessed for the extraction process is 365 MJ/t with MeTHF instead of 284 MJ/t with hexane. Even if a higher amount of energy is provided by recovery from the desolventizer, there is an overconsumption of 81 MJ/t only for the extraction step and a global overconsumption of 85 MJ/t for the whole process (preparation + extraction) with 666 MJ/t for MeTHF instead of 581 MJ/t for hexane. This energy is provided by steam and it represents a total consumption of 304 kg/t with MeTHF instead of 267 kg/t with hexane so at least, an increase of 14% of the energy consumption and therefore of the energetic costs.

#### 2.6.2. Economic Evaluation

This part presents available data on the possible economic impact concerning the replacement of hexane by MeTHF in the rapeseed crushing process. Taking into account the level of investment, energy consumption and employee workforce of several production units, structure costs in a crushing unit were calculated to be around 28 EUR/t [23] seeds, 10–15 EUR/t of seeds for refining costs and refining losses are around the same cost range [24]. In the case of MeTHF, the solvent price currently around 5 EUR/kg (Pennakem, personal communication) is clearly higher than the price of hexane, which is 0.9 EUR/kg. On the basis of a consumption of 0.75 kg of solvent per tonne of crushed seeds an additional cost of around 3 EUR/t can be expected. These costs are not taking into account the possible reductions of the solvent price in case of larger production. Additional steam consumption, see Table 6, was calculated to be around 37 kg/t seeds. Steam price is considered to be at 30 EUR/t so it represents an increase in steam cost of 1.1 EUR/t seeds. Regarding the oil, at least 1% (around 10 kg) more than what is extracted with hexane is extracted with MeTHF. Considering that this oil might possibly be richer in polar compounds, the total gain in the additional oil is evaluated to be around 60% which represents 6 kg of oil per ton of seeds. Nevertheless this additional oil generates an equivalent loss in the amount of cake. As the price of oil is currently around 900 EUR/t [25] and the price of cake is around 350 EUR/t [25], a benefit of 3.30 EUR/t seeds can be realized. Diffusivity of solvent may also have an impact. Diffusivity could have been extrapolated to an increase of the capacity of a factory but it is quite difficult at the present stage of knowledge. The practical consequences are an increase in the capacity of the extractor, which is usually the bottleneck. Assuming that around 5% of capacity is gained, fixed costs are reduced accordingly around 0.8 EUR/t. It is finally a sum of the incremental costs of 4.1 EUR/t against 4.1 EUR/t as increase of productivity and additional products. The hypothesis that no disadvantage in oil acidity is made so it is likely there might be little change in refining costs. Moreover, the use of a new solvent would probably imply a lower tolerance of solvent residues in products for food use compared to what is allowed with hexane. The economic study cannot rely only on technological data but should also take into account sanitary and regulatory aspects that will probably have a huge impact on the process and that are very difficult to anticipate and therefore to assess.

## 3. Experimental Section

### 3.1. Materials and Reagents

Rapeseed, belonging to Astrid variety (Euralis Semences), was provided by the *Centre Technique Interprofessionnel des Oléagineux et du Chanvre industriel* (CETIOM, Pessac, France). *n-*Hexane, 2-methyltetrahydrofuran, methanol, sulfuric acid, sodium chloride, chloroform, methyl acetate, acetic acid, diethyl ether and potassium chloride were of analytical grade and were supplied by VWR International (Darmstadt, Germany).

### 3.2. Lipid Extraction

#### 3.2.1. Conventional Soxhlet Procedure

**Figure 5:**
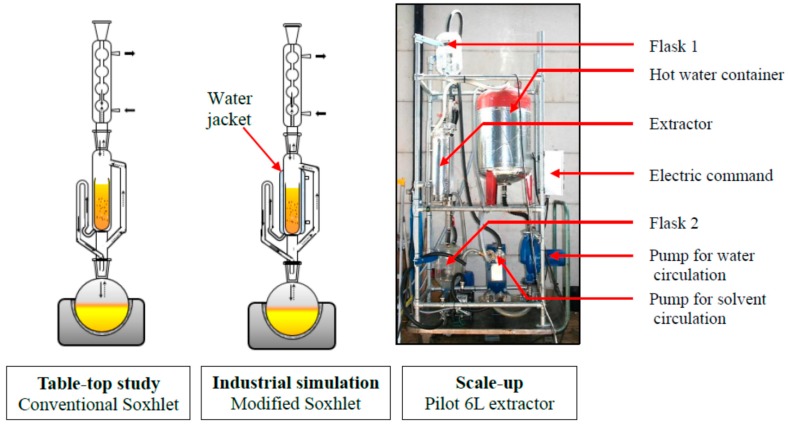
Experimental apparatus.

Rapeseed was coarsely ground less than 30 min before extraction. The moisture level of seeds was determined using a MB35 moisture analyzer (Ohaus, Nänikon, Switzerland). Oils were isolated from seeds by means of Soxhlet extraction [26]. According to official method (ISO nr 659) [27] 30 grams of coarsely ground rapeseeds was weighed and transferred into a 30 mm × 100 mm cellulose thimble (Macherey-Nagel, Düren, Germany), which was plugged with cotton in order to avoid transfer of sample particles to the distillation flask. They were then placed in the extraction chamber of a 200 mL Soxhlet apparatus, see Figure 5, fitted with a condenser, which was placed on a 500 mL distillation flask containing 300 mL of solvents (*n-*hexane or MeTHF, respectively). Samples were extracted under reflux with *n-*hexane and MeTHF during 4 h (18–22 cycles/h). Thereafter, the cellulose thimble was cooled to room temperature in a desiccator and its content was then ground before being loaded again in the cellulose cartridge. The described procedure was thus repeated during 2 h until a total extraction of 8 h (4 + 2 + 2 h). After the extraction the content of the distillation flask was evaporated under reduced pressure. The flask was then weighted and this operation repeated until the difference between two consecutive weights was less than 10% (*w*/*w*). The weight of the rapeseed oil was determined and then used for calculating the yield of extracted oil. All extractions were performed in triplicate and the mean values were reported.

Results were obtained by gas chromatographic analysis in order to know the yields of extracted oils, *i.e.*, the lipid content, as explained in point 3.5. The yield of extracts obtained by GC after trans-methylation of fatty acids was expressed as a percentage of the weight of extracted oil obtained after extraction relative to the weight of dry rapeseeds used for extraction, as described hereinafter,

(8) $$Yield\;of\;Extracts\left(\%\right)=\frac{Weight\;of\;oil}{Weight\;of\;rapeseeds\;(dry\;materials)}\times 100$$

#### 3.2.2. Modified Soxhlet Procedure

In order to study the industrial process and the transposition of scale, a laboratory scale of the pilot scheme was achieved. Thereby, a special Soxhlet apparatus was manufactured by Legallais (Montferrier-sur-Lez, France) with a water jacket around the extraction chamber to set the temperature of the extraction as shown in Figure 5. 30 grams of matrix (rapeseeds previously pressed using a Komet press CA59G3 (IBG Monforts Oekotec, Mönchengladbach, Germany), moisture content of 7.74%, lipid content of 47%) were weighed and introduced in a 30 mm × 100 mm cellulose thimble (Macherey-Nagel). Press cake was extracted during 2 h with 300 mL *n-*hexane and 300 mL MeTHF respectively. The residual oil in the matrix was then determined using the conventional Soxhlet extraction procedure described in Section 2.2.1.

### 3.3. Kinetics Studies

Kinetics study was performed on 30 g rapeseed flakes macerated at 55 °C with 150 mL of *n-*hexane and 150 mL of MeTHF respectively. This ratio was selected to reproduce the conditions inside the extraction chamber that contains 150 mL solvent once completely filled. In order to follow the kinetic, approximately 1 mL of sample was collected from the flask after 1, 3, 5, 10, 15, 20 and 30 min, then the same volume of sample was withdrawn every 30 min during 2 h of extraction. The kinetics were established with mass difference. Each sample was weighed and was then inerted with nitrogen at 40 °C in a block heater with sample concentrator to evaporate the solvent in order to know the percentage of extract thanks to the mass of solvent and the mass of extract inside the sample. This percentage is then related to the total mass of extract knowing the initial mass of total used solvent (linked to the volume of 300 mL by the density) and taking into account the mass of solvent and extract removed for the sampling.

### 3.4. Pilot Scale Procedure

A pilot scale study was conducted with hexane and MeTHF on press cake (see Section 3.2.2.) using a 6 L extractor (CREOL, Pessac). In this device (Figure 5) the solvent is forced to percolate through a layer of solid (400 mm) to continuously wash the matrix and solubilize oil contained into seeds. The miscella (containing solvent and extracted oil) is moved by a pump which circulates continuously through a heat exchanger. Trials were realized on 2 kg matrix with 1.5 times more solvent (*m*/*m*). This ratio was selected as it was calculated to simulate what is done in industrial plants with counter-current extractors [28,29]. Experiments were performed by doing five washings of 30 min each using “clean” solvent. Oil content in solid residue was then determined using the conventional Soxhlet extraction procedure described in Section 3.2.1.

### 3.5. Chromatographic Analysis

Fatty acids methyl esters (FAMEs) were separated, identified and quantified by gas chromatography coupled with flame ionization detector (GC-FID). Samples were prepared from extracted oils using acid-catalyzed trans-methylation [30]. One mL methanolic sulfuric acid (5% *v*/*v*) was added to a specific amount of extracted oils and internal standard. The mixture was then heated at 85 °C for 90 min and then removed from heater. One point five mL of sodium chloride (0.9%) solution and 1 mL of *n*-hexane were added afterwards. The flask was stoppered and shaken vigorously during 30 s before centrifugation at 4000 rpm for 2 min. A small amount of the organic layer was removed and transferred into a vial before being injected directly in a gas chromatography.

Analyses were performed by a 7820A GC system (Agilent technologies, Santa Clara, CA, USA) equipped with a FID detector and auto-sampler. Gas chromatography was performed by a BD-EN14103 capillary column (30 m × 0.32 mm × 0.25 µm) using helium as a carrier gas at the velocity of 33 cm/s. 2 µL of various samples were injected in split mode (split ratio: 1:20) at 250 °C. The oven temperature program was operated as follows: initial temperature at 50 °C for one minute, increasing at a rate of 20 °C/min to 180 °C and at a rate of 2 °C/min from 180 to 230 °C, held isothermally at 230 °C for 10 min. Data were collected with Agilent EZChrom Elite software. FAMEs were identified in comparison with purified FAME standards (Sigma Co., St. Louis, MO, USA). Quantification was performed thanks to internal calibration. The internal standard was glyceryl tripheptanecanoate (Sigma, Co.).

Lipids were detected by charring and quantified using a CAMAG 3 TLC scanning densitometer (CAMAG, Muttenz, Switzerland) with identification of the classes against known polar and neutral lipid standards. Typically, lipid extract was loaded as a spot onto 20 cm × 10 cm silica gel 60 F254 HPTLC plates (Merck KGaA, Darmstadt, Germany) using an ATS 5 automatic TLC sampler (Camag, Muttenz, Switzerland). Plates were then developed in an ADC2 automatic developing chamber (CAMAG) using first a methyl acetate/isopropanol/chloroform/methanol/KCl (0.25% solution) (25:25:25:10:9) mixture running to a height of 5.5 cm from the origin and then a n-hexane/diethyl ether/glacial acetic acid mixture (70:30:2) to a height of 8.5 cm from the origin. After drying, the plate was dipped for 6 s in a modified CuSO_4_ reagent (20 g CuSO_4_, 200 mL methanol, 8 mL H_2_SO_4_, and 8 mL H_3_PO_4_) then heated at 141 °C for 30 min on a TLC plate heater and finally scanned using a TLC Scanner 3 with WinCATs software (CAMAG). The densitometry data are reported as values which are expressed as percent of lipid class in total rapeseed lipids.

Other constitutes in extracted oils were quantitatively and qualitatively analyzed according to respective norms: NF EN ISO 12,228 for sterols [31], and NF EN ISO 9936 for tocopherol and tocotrienol [32].

### 3.6. Computational Methods

Solubility parameters of solvents have been studied by means of HSP [2] theoretical prediction and COSMO-RS [3,4].

#### 3.6.1. Hansen Solubility Parameters

HSP provides a convenient and efficient way to characterize solute-solvent interactions according to the classical “like dissolves like” rule. HSP was based on the concept that the total cohesive energy density is approximated by the sum of the energy densities required to overcome atomic dispersion forces (δ_d_^2^), molecular polar forces arising from dipole moments (δ_p_^2^) and hydrogen-bonds (exchange of electrons, proton donor/acceptor) between molecules (δ_h_^2^), as given in the following equation:

(9) δ_total_^2^ = δ_d_^2^ + δ_p_^2^ + δ_h_^2^

where δ_total_ is the Hansen total solubility parameter, which now consists of three Hansen solubility parameters in terms of dispersion (δ_d_), polar (δ_p_) and hydrogen-bonding (δ_h_).

Typically, the more similar the two δ_total_ are, the greater the affinity between solute and solvent. The chemical structures of the solvents and solutes discussed in this article could be mutually transformed by JChemPaint version 3.3 (GitHub Pages, San Francisco, CA, USA) to their simplified molecular input line entry syntax (SMILES) notations, which were subsequently used to calculate the solubility parameters of various solvents and constituents in extracted oil. These solubility parameters were further modeled to a frequently-used two dimensional HSP sphere for better visualizing the solute/solvent interaction because of insignificant differences between δ_d_ s (HSPiP Version 4.0, Hansen-Solubility, Hørsholm, Denmark).

#### 3.6.2. COSMO-RS Calculations

COSMO-RS is a quantum chemistry solvation model based on the prediction of chemical potential of a substance in the liquid phase [3,4]. Calculation of the relative solubility of major compounds of rapeseed oil in various solvents was made by implementing this COSMO-RS model in COSMOtherm software (C30 1201, COSMOlogic, Leverkusen, Germany). The relative solubility is calculated from the following equation [33]:

(10) $${\text{log}}_{10}(x_{j})={\text{log}}_{10}\left[\frac{exp\left({\mu_{j}}^{\text{pure}}-{\mu_{j}}^{\text{solvent}}-∆G_{j,fusion}\right)}{RT}\right]$$

with ${\mu_{j}}^{\text{pure}}$: chemical potential of pure compound *j*; ${\mu_{j}}^{\text{solvent}}$: chemical potential of j at infinite dilution; $∆G_{j,fusion}$: free energy of fusion of *j*; $x_{j}$: solubility of *j*.

Relative solubility is always calculated in infinite dilution. The logarithm of the best solubility is set to 0 and all other solvents are given relatively to the best solvent. A solvent with a log_10_ (x-solub) value of −1.00 yields a solubility which is decreased by a factor 10 compared to the best solvent.

## 4. Conclusions

The aim of the present study was to investigate the replacement of hexane for extraction of vegetable oil by bio-based, non-toxic and biodegradable solvent. A solvent selection using HSP and COSMO-RS simulation tools as well as theoretical properties of candidate solvents showed that MeTHF is the most suitable solvent to replace hexane due to its properties to dissolve not only triglycerides but also the other classes of lipids such as sterols, tocopherols and tocotrienols. The experimental laboratory study confirms these theoretical simulations. We compared hexane and MeTHF in terms of yield, selectivity, chemical composition, and kinetics and diffusion studies. Pilot plant experimentation as well as energy and economic evaluation of the process prove that MeTHF could be a potential industrial alternative to hexane.
